# Enhanced Removal of Cd(II) Ions from Aqueous Media via Adsorption on Facilely Synthesized Copper Ferrite Nanoparticles

**DOI:** 10.3390/molecules29153711

**Published:** 2024-08-05

**Authors:** Nada S. Al-Kadhi, Maram T. Basha

**Affiliations:** 1 Department of Chemistry, College of Science, Princess Nourah bint Abdulrahman University, Riyadh 11671, Saudi Arabia; nsalkadhi@pnu.edu.sa; 2 Department of Chemistry, College of Science, University of Jeddah, Jeddah 21589, Saudi Arabia

**Keywords:** nanomaterials, adsorptive process, potentially toxic elements, analytical parameters

## Abstract

In this study, magnetic copper ferrite (CuFe_2_O_4_) nanoparticles were synthesized via the Pechini sol-gel method and evaluated for the removal of Cd(II) ions from aqueous solutions. PF600 and PF800 refer to the samples that were synthesized at 600 °C and 800 °C, respectively. Comprehensive characterization using FTIR, XRD, FE-SEM, HR-TEM, and EDX confirmed the successful formation of CuFe_2_O_4_ spinel structures, with crystallite sizes of 22.64 nm (PF600) and 30.13 nm (PF800). FE-SEM analysis revealed particle diameters of 154.98 nm (PF600) and 230.05 nm (PF800), exhibiting spherical and irregular shapes. HR-TEM analysis further confirmed the presence of aggregated nanoparticles with average diameters of 52.26 nm (PF600) and 98.32 nm (PF800). The PF600 and PF800 nanoparticles exhibited exceptional adsorption capacities of 377.36 mg/g and 322.58 mg/g, respectively, significantly outperforming many materials reported in the literature. Adsorption followed the Langmuir isotherm model and pseudo-second-order kinetics, indicating monolayer adsorption and strong physisorption. The process was spontaneous, exothermic, and predominantly physical. Reusability tests demonstrated high adsorption efficiency across multiple cycles when desorbed with a 0.5 M ethylenediaminetetraacetic acid (EDTA) solution, emphasizing the practical applicability of these nanoparticles. The inherent magnetic properties of CuFe_2_O_4_ facilitated easy separation from the aqueous medium using a magnet, enabling efficient and cost-effective recovery of the adsorbent. These findings highlight the potential of CuFe_2_O_4_ nanoparticles, particularly PF600, for the effective and sustainable removal of Cd(II) ions from water.

## 1. Introduction

Water contamination with potentially toxic elements arises from both natural and anthropogenic sources. Natural leaching from the Earth’s crust can introduce metals into waterways, but human activities such as mining, industrial processes, agricultural runoff, and improper waste disposal significantly exacerbate the levels of these contaminants [1,2,3]. Industrial effluents, particularly from facilities that process or use potentially toxic elements in their operations, are a major contributor to contamination, releasing large quantities of metals into water bodies without adequate treatment. Urban runoff and obsolete plumbing systems can also introduce significant amounts of heavy metals into water supplies [4,5,6]. Potentially toxic elements like lead, mercury, arsenic, and cadmium are toxic environmental pollutants. In ecosystems, they can bioaccumulate in the food chain, leading to detrimental effects on wildlife, including reproductive failure and acute toxicity. For humans, chronic exposure to potentially toxic elements, even at low levels, can result in devastating health issues, ranging from kidney damage and bone fragility to neurological disorders and an increased risk of cancer [7,8,9]. The persistence and non-degradable nature of potentially toxic elements make them a persistent threat to environmental and human health. Cadmium (Cd) is especially notorious for its high toxicity to human health. Exposure to Cd(II) ions, even in small amounts, can lead to serious health problems such as kidney damage, and bone demineralization (Itai-Itai disease), and is classified as a human carcinogen. Cadmium is also known to cause defects in renal tubule function, accumulate in renal tissues, and be associated with hypertension and cardiovascular diseases [10,11,12]. There are numerous strategies for removing heavy metals from contaminated water, including chemical precipitation [13], ultrafiltration [14], reverse osmosis [15], electrodialysis [16], and adsorption [17]. Chemical precipitation is a simple method, particularly suitable for removing large amounts of metals from wastewater. This method can generate large volumes of sludge that require proper disposal, and it may not be effective for treating very low concentrations of potentially toxic elements. Ultrafiltration can effectively remove colloidal particles and high-molecular-weight substances, providing a high level of metal removal efficiency. This process can be energy-intensive and costly due to the need for frequent membrane cleaning and replacement to prevent fouling. Reverse osmosis offers high efficiency in removing dissolved potentially toxic elements, producing high-purity water. It is effective even for very low concentrations of contaminants. This process is expensive, both in terms of capital investment and operational costs, and it generates a significant amount of concentrated brine that needs to be managed. Electrodialysis is effective for the selective removal of ionic species, allowing for the recovery of valuable metals. It operates at lower energy costs compared to reverse osmosis. The process requires complex and expensive equipment, and it can be less effective in the presence of non-ionic contaminants. Adsorption is often preferred due to its effectiveness, efficiency, and cost advantages. It is flexible, easy to operate and maintain, and can be used to target specific contaminants. The performance of adsorption is highly dependent on the adsorbent material, which can be costly [18,19]. Nano-metal oxides have emerged as highly effective adsorbents due to their large surface areas and high reactivity. Nanoscale particles offer enhanced kinetics and a greater surface area, making them excellent candidates for capturing and immobilizing heavy metal ions [20,21]. Recent studies have demonstrated the effectiveness of ferrite-based nanomaterials in the removal of heavy metals. For instance, nickel ferrite nanoparticles have shown adsorption capacities of 21.30 mg/g for Cr(VI), 19.21 mg/g for Pb(II), and 21.11 mg/g for Cd(II), highlighting their potential as cost-effective adsorbents [22]. Similarly, cobalt and nickel ferrite graphene nanocomposites have exhibited high adsorption capacities, with equilibrium values of 142.8 mg/g and 111.1 mg/g for Pb(II), and 105.26 mg/g and 74.62 mg/g for Cd(II), respectively, underscoring their suitability for environmental remediation [23]. The Pechini sol-gel approach is valuable for preparing nano-metal oxides because it allows for precise control over the material’s chemical composition and structural properties. This method involves the use of a chelating agent to bind metal ions, which are then incorporated into a polymer network formed by a cross-linking agent, leading to highly uniform and pure nanooxides [24,25]. The versatility and effectiveness of the Pechini method for synthesizing complex oxide materials are well documented in the literature. Chandrani et al. used two methods, modified Pechini and green synthesis, to create cobalt ferrite nanoparticles [26]. The modified Pechini method involves metal salts, ethylene glycol, and citric acid, while the green synthesis uses ginger root and cardamom seed extracts. The synthesized nanoparticles, confirmed by XRD to have a spinel structure with sizes of 14–20 nm, were characterized using various techniques and tested for photocatalytic properties with phenol red dye. This research innovatively uses the Pechini sol-gel method to synthesize magnetic CuFe_2_O_4_ nanoparticles, employing tartaric acid as a chelating agent and propylene glycol as a cross-linker. This approach not only ensures the homogeneity and purity of the nanoparticles but also enhances their stability and adsorption capacity. The focus on cadmium is due to its significant presence in industrial wastewater and its severe health impacts, making it crucial to develop efficient methods for its removal. The synthesized nanoparticles demonstrate exceptional adsorption properties, offering a promising solution to mitigate the impact of cadmium pollution and contribute significantly to safer and cleaner water resources. Additionally, copper ferrite nanoparticles (CuFe_2_O_4_) were selected for this study due to their excellent magnetic properties, high surface area, and strong affinity for Cd(II) ions, which make them efficient adsorbents. The stability and ease of separation using an external magnetic field further justify their selection for the effective removal of Cd(II) ions from aqueous solutions.

## 2. Results and Discussion

### 2.1. Synthesis and Characterization of Copper Ferrite Nanoparticles

**Scheme 1:**
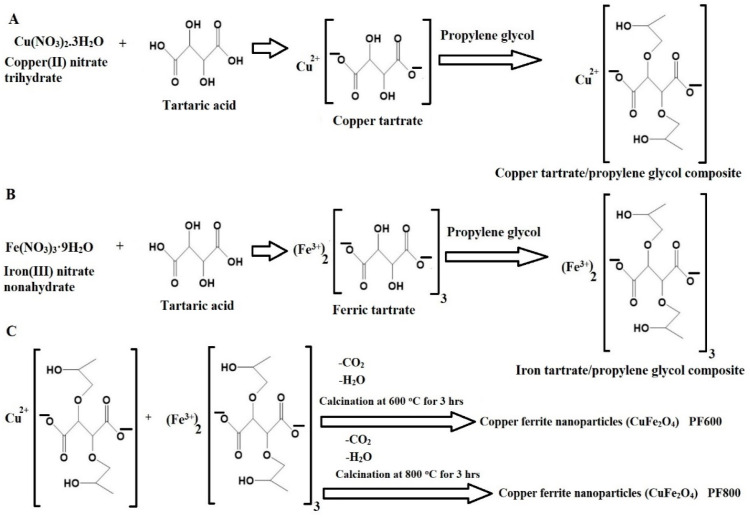
Production of copper ferrite nanoparticles through the Pechini sol-gel process.

Scheme 1 illustrates the Pechini sol-gel method to synthesize CuFe_2_O_4_ nanoparticles. Copper(II) nitrate trihydrate and iron(III) nitrate nonahydrate react with tartaric acid to form tartrate complexes as shown in Scheme 1A,B, which are polymerized with propylene glycol [27,28,29,30]. The resulting composites are calcined at 600 and 800 °C to remove the organic part and form copper ferrite nanoparticles as shown in Scheme 1C. Temperatures below 600 °C do not form the crystalline facies of copper ferrite, as these lower temperatures are insufficient to completely remove organic materials during the preparation process.

The synthesis of CuFe_2_O_4_ nanoparticles using the Pechini sol-gel method is an environmentally friendly approach because it utilizes biodegradable, inexpensive, and non-toxic chemicals. The cost of producing CuFe_2_O_4_ nanoparticles is approximately 0.10 $ per gram.

**Figure 1:**
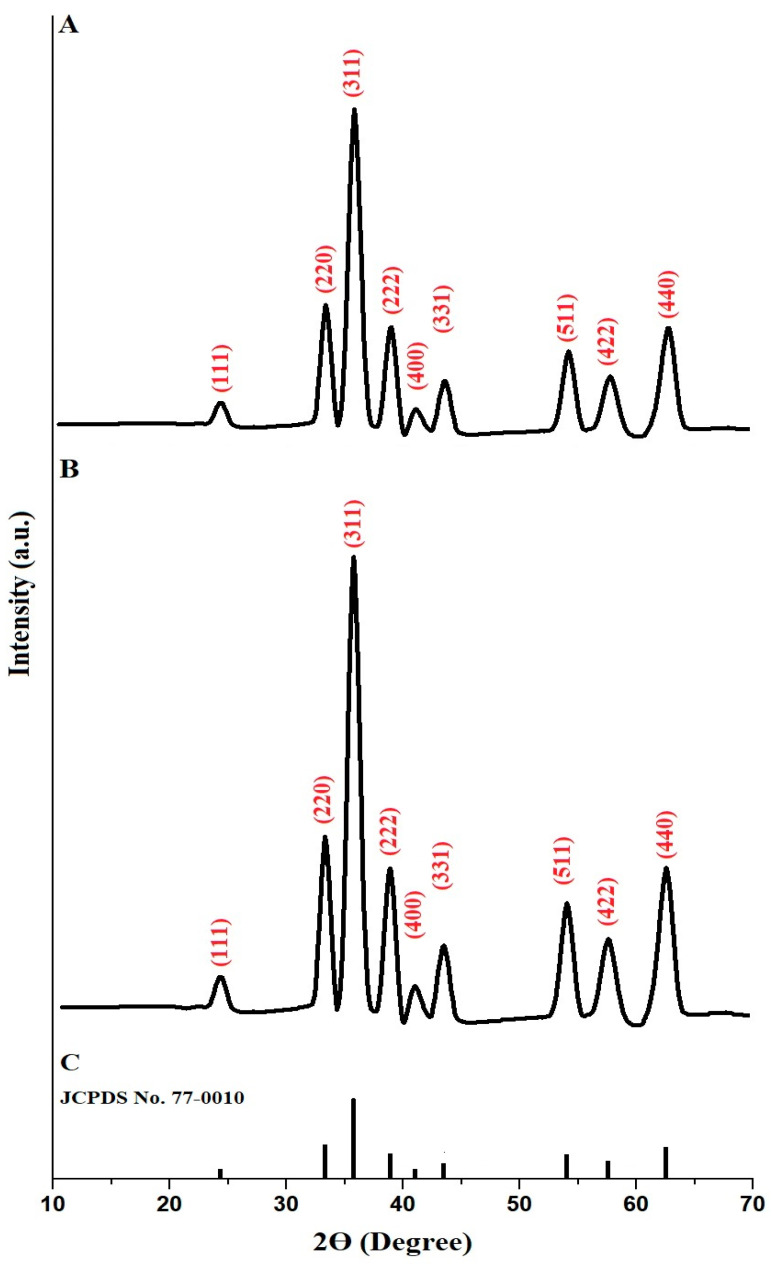
XRD analysis of the PF600 (**A**) and PF800 (**B**) products. Standard JCPDS No. 77-0010 (**C**).

The crystallinity of copper ferrite samples, identified as PF600 and PF800, was analyzed using XRD, with findings illustrated in Figure 1A, and 1B, respectively. Based on the standard JCPDS No. 77-0010 (Figure 1C), both samples exhibited distinct diffraction peaks that aligned with the cubic spinel structure typical of copper ferrite (CuFe_2_O_4_) [31]. The diffraction peaks at 2θ angles of 24.28°, 33.19°, 35.66°, 38.79°, 40.89°, 43.37°, 53.93°, 57.33°, and 62.38° correspond to the miller indices (111), (220), (311), (222), (400), (311), (511), (422), and (440) of CuFe_2_O_4_, respectively. Besides, the average crystal size, which was determined by Scherrer equation [32], of the PF600 and PF800 products was found to be 22.64 and 30.13 nm, respectively. Hence, the changes due to calcination are thus evident in the differences in crystallite sizes and the sharpness of the diffraction peaks, confirming that higher calcination temperatures enhance the crystal growth of CuFe_2_O_4_ nanoparticles and produce large crystallite sizes.

**Figure 2:**
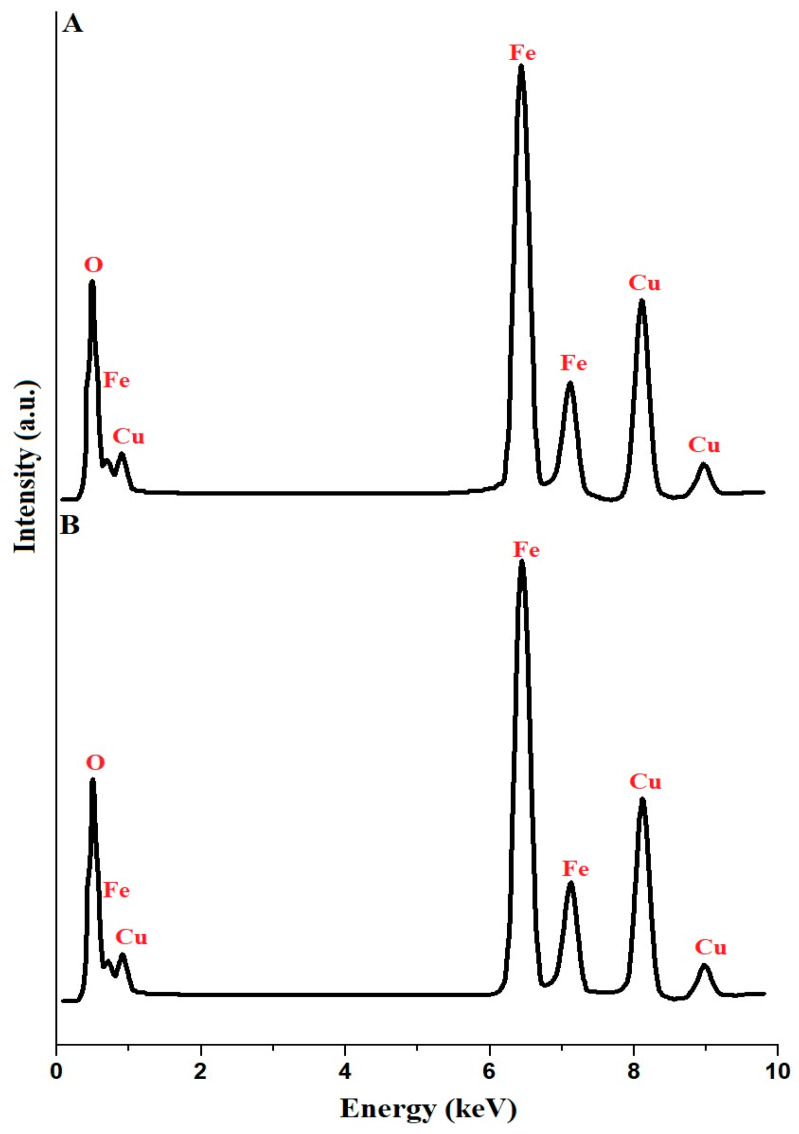
EDX analysis of the PF600 (**A**) and PF800 (**B**) products.

In Figure 2A,B, the EDX analysis results for the copper ferrite samples, identified as PF600 and PF800, are meticulously presented. The analysis confirms the presence of the essential components for CuFe_2_O_4_ in both samples: copper (Cu), iron (Fe), and oxygen (O). The weight percentage composition of the PF600 sample is reported as Cu: 25.91%, Fe: 47.12%, and O: 26.97%. Besides, the weight percentage composition of the PF800 sample is reported as Cu: 24.11%, Fe: 46.93%, and O: 28.96%. These percentages demonstrate that the composition of Cu, Fe, and O in the samples is in close agreement with the theoretical composition of copper ferrite, symbolically denoted by the ratio Fe:Cu:O of 2:1:4 [31].

**Figure 3:**
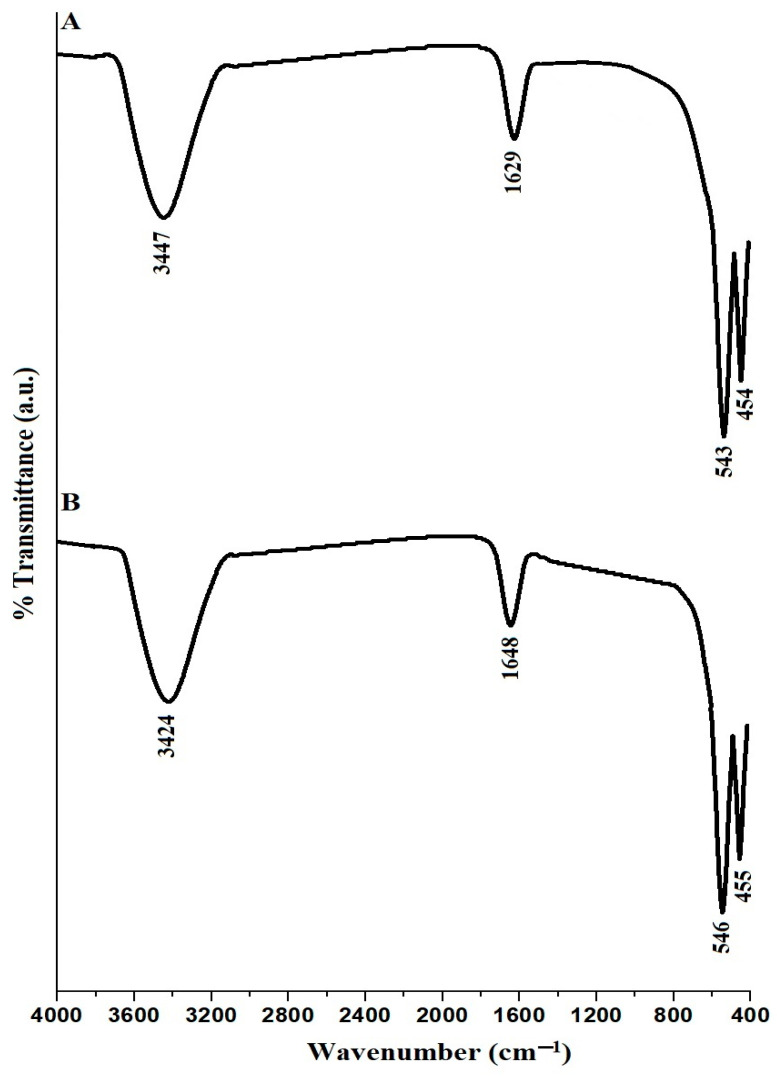
FTIR analysis of the PF600 (**A**) and PF800 (**B**) products.

The FTIR examination of CuFe_2_O_4_ nanoparticles, labeled PF600 and PF800 in Figure 3A,B, reveals unique FTIR spectra for CuFe_2_O_4_ nanoparticles. The observation of broad bands at 3447 cm^−1^ for PF600 and 3424 cm^−1^ for PF800 is typically associated with O-H stretching vibrations, which suggests the existence of hydroxyl groups on the nanoparticle surfaces or the adsorption of water molecules. Additionally, the absorption bands at 1629 cm^−1^ for PF600 and 1648 cm^−1^ for PF800 are believed to arise from O-H bending vibrations. Both PF600 and PF800 samples display two distinct bands, approximately at 543 and 454 cm^−1^ for PF600, and 546 and 455 cm^−1^ for PF800, respectively. These bands signify the vibrational frequencies of metal-oxygen bonds located in the octahedral (Fe-O) and tetrahedral (Cu-O) configurations, which are indicative of the CuFe_2_O_4_ spinel structure [31,33].

**Figure 4:**
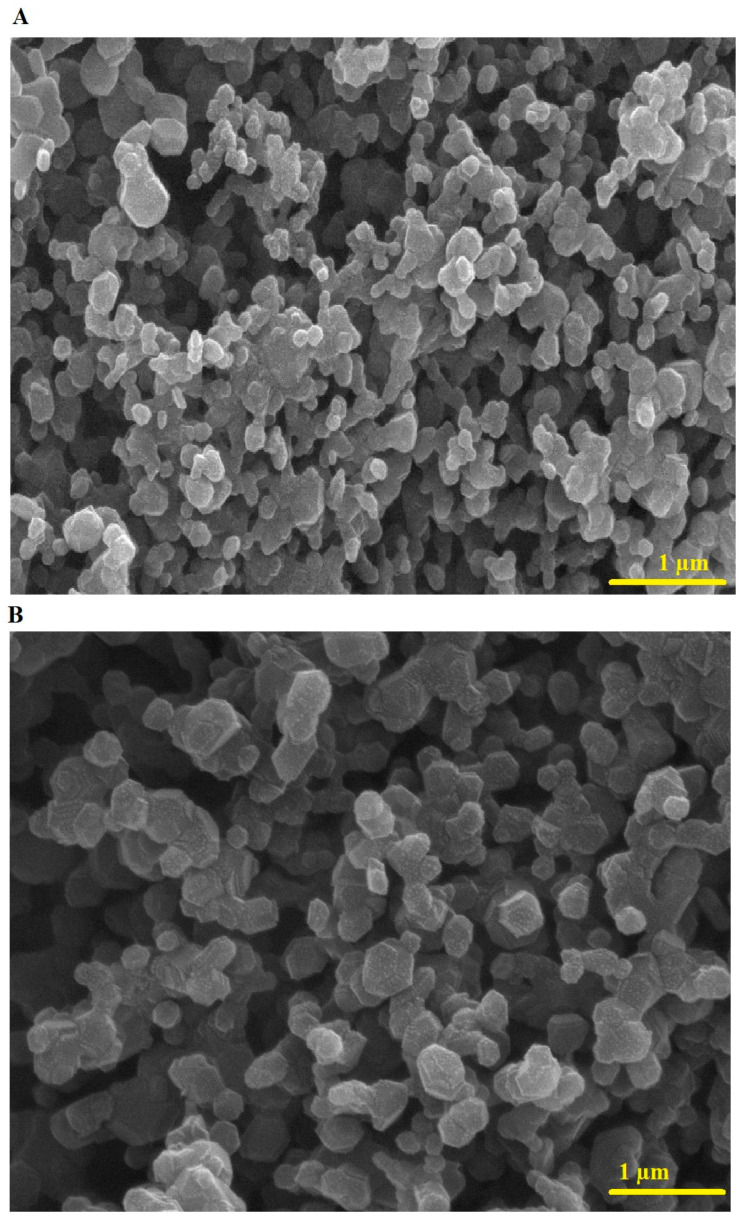
FE-SEM analysis of the PF600 (**A**) and PF800 (**B**) samples.

Figure 4A,B represent the FE-SEM images of the PF600 and PF800 samples, respectively. The PF600 and PF800 samples consist of spherical and irregular shapes with average diameters of 154.98 and 230.05 nm, respectively. The crystallite size, determined by XRD, refers to the size of single crystalline domains (22.64 nm for PF600 and 30.13 nm for PF800), while the nanoparticle size, measured by FE-SEM, includes the entire particle, which may consist of multiple crystallites (154.98 nm for PF600 and 230.05 nm for PF800). This explains why the observed nanoparticle sizes are significantly larger than the crystallite sizes.

The presence of Cu(II) ions in the octahedral sites introduces Jahn-Teller distortions, which are characteristic of these ions due to their electronic configuration. These distortions lead to tetragonal elongation or compression in the lattice, affecting the local cationic distribution. The Jahn-Teller effect not only impacts the structural characteristics but also influences the magnetic and adsorption properties of the nanoparticles. The distortions can enhance the magnetic interactions between the Fe(III) ions in the tetrahedral and octahedral sites, which may improve the magnetic separation efficiency of the nanoparticles. Moreover, the surface morphology and particle size distribution, as revealed by FE-SEM, show irregular shapes with varying diameters, which can be attributed to these structural distortions.

**Figure 5:**
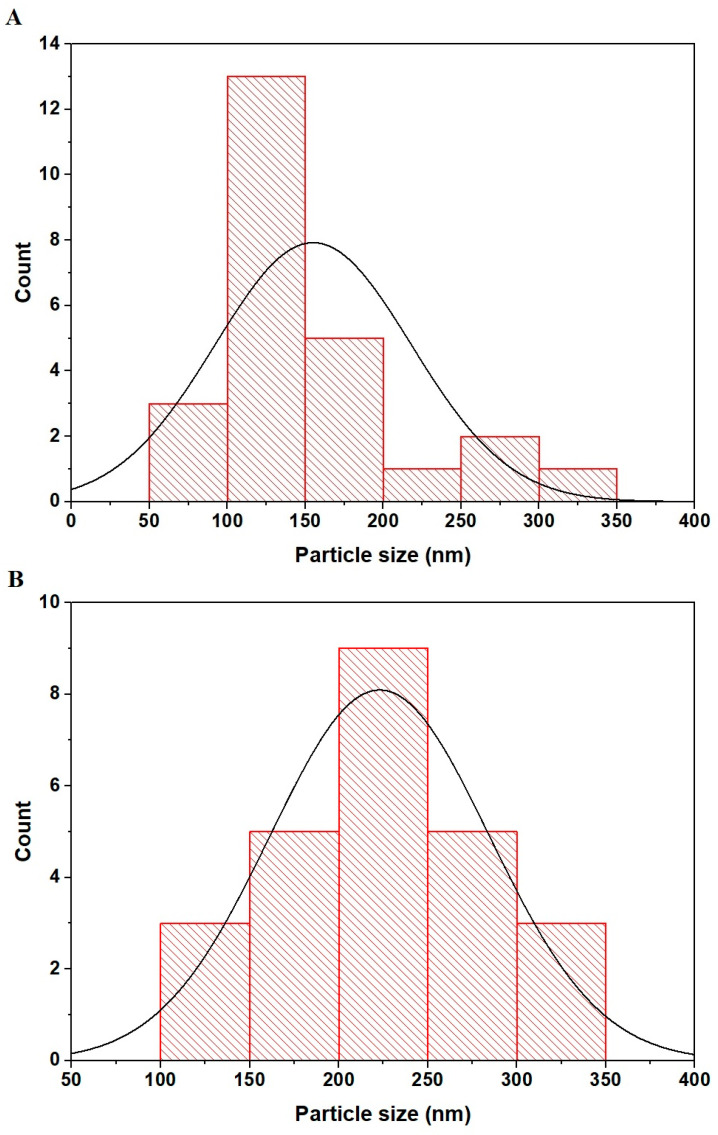
FE-SEM histograms of the PF600 (**A**) and PF800 (**B**) samples.

In Figure 5A,B, FE-SEM histograms for the PF600 and PF800 CuFe_2_O_4_ samples are depicted, respectively. The results were obtained from SEM images using Image J software v.No. 1.53f. In the PF600 sample, as shown in histogram A, a concentration of particle sizes is seen around the 100 to 150 nm range with a peak count near 150 nm, indicating a more uniform particle size distribution. For the PF800 sample, represented by histogram B, particle sizes are distributed across a broader range, with significant counts between 150 and 300 nm and the peak shifted to around 200 nm, suggesting a wider variation in particle sizes.

**Figure 6:**
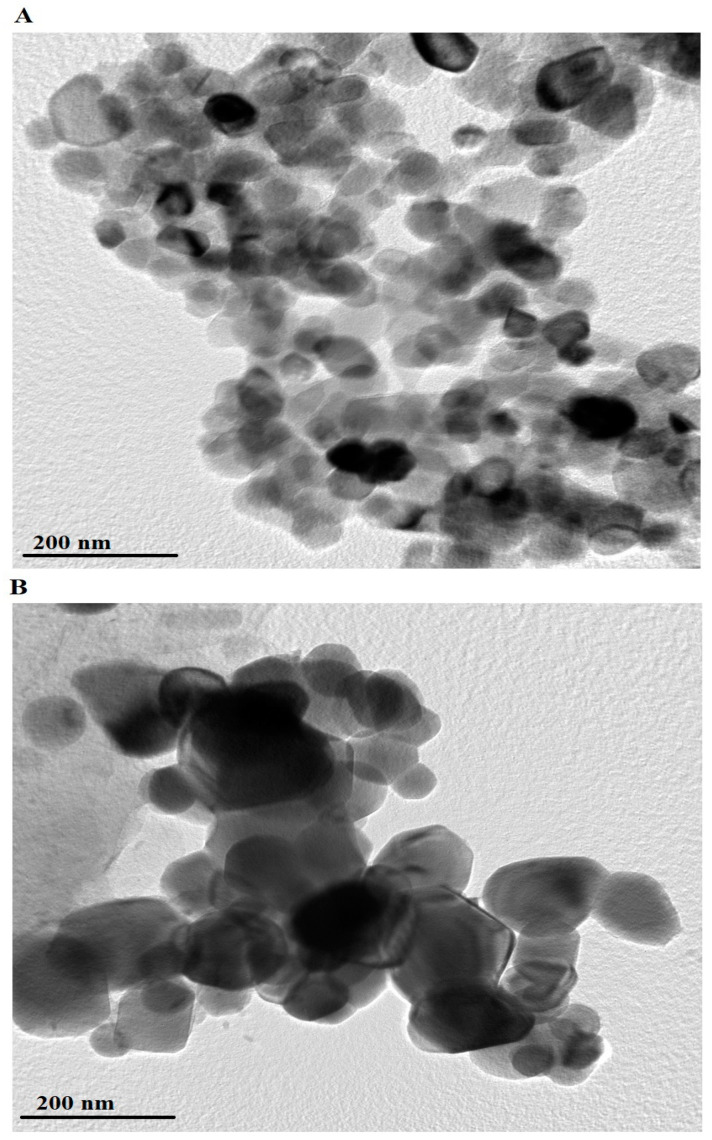
HR-TEM analysis of the PF600 (**A**) and PF800 (**B**) products.

HR-TEM images of the PF600 and PF800 products are shown in Figure 6A,B, respectively. The PF600 sample (Figure 6A) displays nanoparticles with an average diameter of 52.26 nm. The particles in the PF600 sample appear to be irregularly shaped and are aggregated into clusters. In contrast, the PF800 sample (Figure 6B) consists of nanoparticles with a larger average diameter of 98.32 nm. The nanoparticles in the PF800 sample are more uniformly distributed and exhibit a more defined spherical shape. The discrepancy between crystal size determined by XRD and TEM arises because XRD measures average crystallite size, considering internal strain and peak broadening, while TEM provides direct visualization of individual particle size and shape, including agglomeration and surface effects.

### 2.2. Removal of Cd(II) Ions from Aqueous Media

#### 2.2.1. Influence of pH

**Figure 7:**
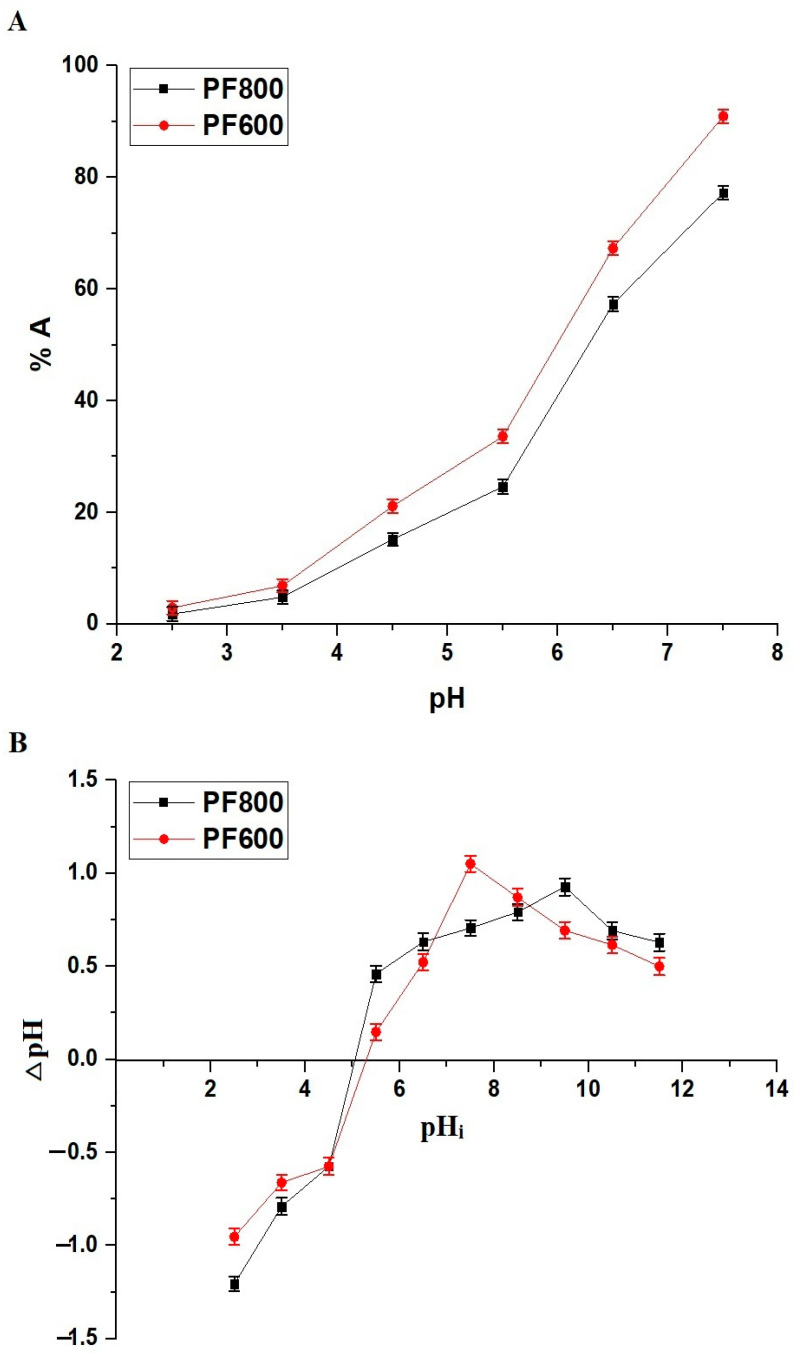
The adsorption percentage of cadmium ions on the PF600 and PF800 products throughout a range of pH values (**A**). The point of zero charge (pH_PZC_) of the PF600 and PF800 products (**B**).

Figure 7A displays the varying percentages of Cd(II) ion adsorption by the PF600 and PF800 samples at different pH levels. A noticeable increase in adsorption percentage is seen as the pH moves from 2.5 to 7.5. At a pH of 7.5, the adsorption percentages for cadmium ions on the PF800 and PF600 samples are recorded at 77.29 and 90.96%, respectively. The pH range was limited to 7.5 to avoid precipitation of cadmium as Cd(OH)_2_, which typically occurs at higher pH values, thus interfering with the adsorption study. In comparison to previous studies, our results show a significant influence of pH on the adsorption capacity of CuFe_2_O_4_ nanoparticles. For instance, the maximum adsorption percentages observed in this study at pH 7.5 (90.96% for PF600 and 77.29% for PF800) surpass the adsorption efficiencies reported for chitosan-coated perlite beads and polyaniline grafted chitosan, which achieved lower efficiencies at similar pH conditions [34,35].

##### Adsorption Mechanism

**Scheme 2:**
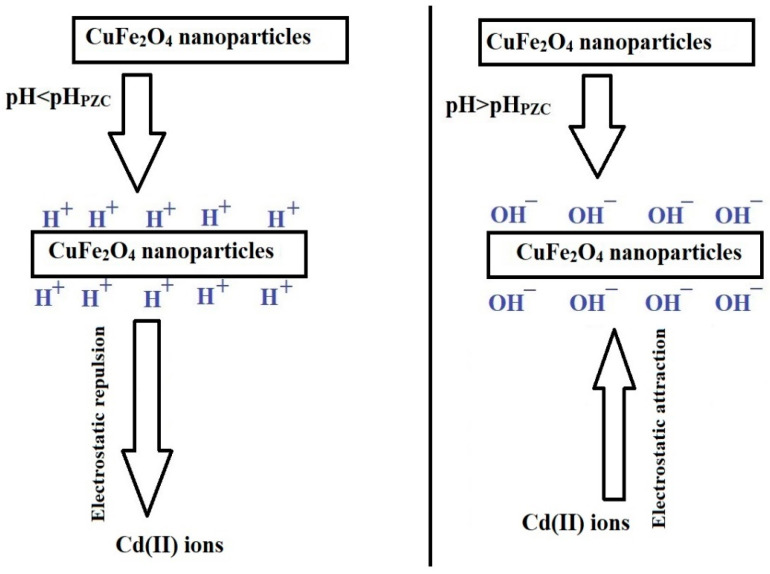
Removal mechanism of cadmium ions by the synthesized copper ferrite nanoparticles.

This adsorption is significantly affected by the pH, which is linked to the adsorbents’ point of zero charge (pH_PZC_). As indicated in Figure 7B, the PF800 and PF600 adsorbents have pH_PZC_ values of 5.05 and 5.29, respectively. This pattern indicates that when the pH exceeds the pH_PZC_, the adsorbent surface takes on a more negative charge, thus improving the adsorption of Cd(II) ions because of electrostatic attraction, as demonstrated in Scheme 2 [30]. Contrarily, at pH levels below the pH_PZC_, the surface of the adsorbents becomes positively charged, which leads to a decrease in the adsorption of Cd(II) ions as a result of electrostatic repulsion [30,36,37].

**Figure 8:**
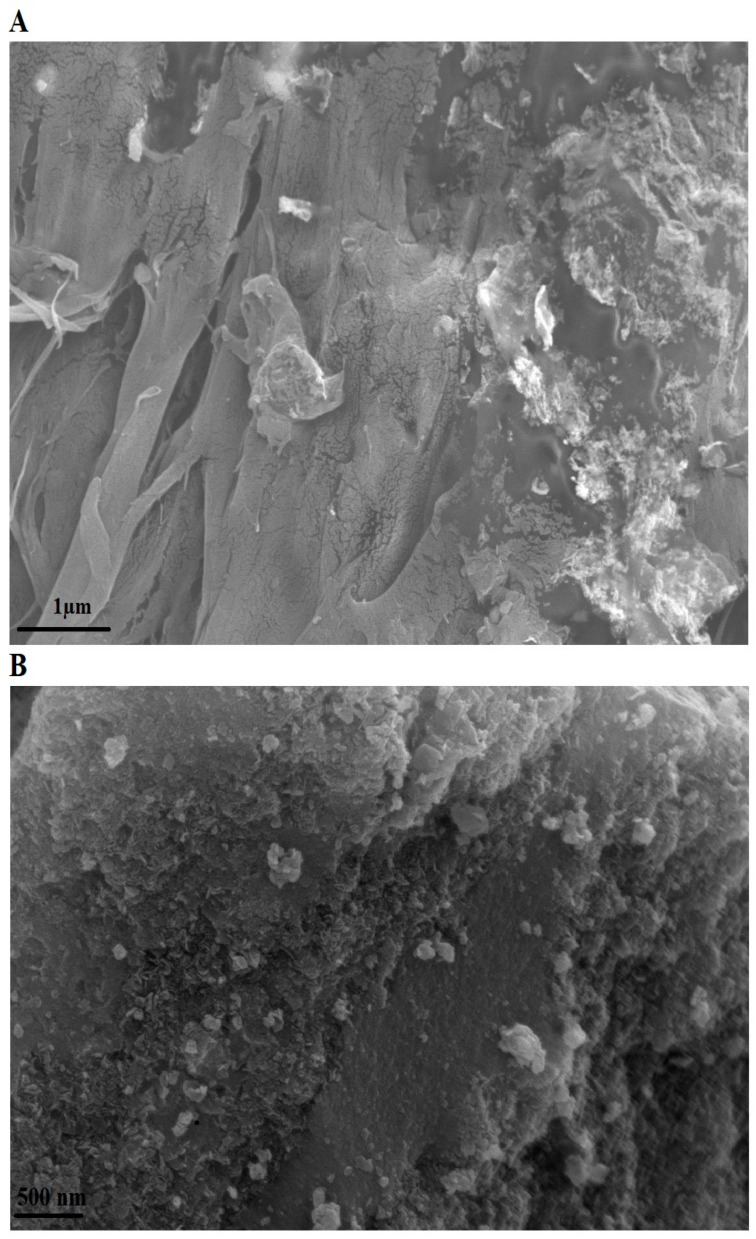
FE-SEM image of the PF600 sample after Cd(II) adsorption at magnifications of 15,000× (**A**) and 3000× (**B**).

The surface morphology of the PF600 sample (as an illustrative example) after Cd(II) adsorption was examined using FE-SEM at magnifications of 15,000× and 30,000×, as shown in Figure 8A and 8B, respectively. Post-adsorption, the FE-SEM image reveals a noticeable change in the surface texture of the nanoparticles. The previously observed irregular shapes and smooth surfaces (Figure 4A) have been replaced by a more aggregated and rougher surface, indicating the successful adsorption of Cd(II) ions. These changes suggest the presence of Cd(II) ions on the surface of the nanoparticles, further supporting the adsorption mechanism.

**Figure 9:**
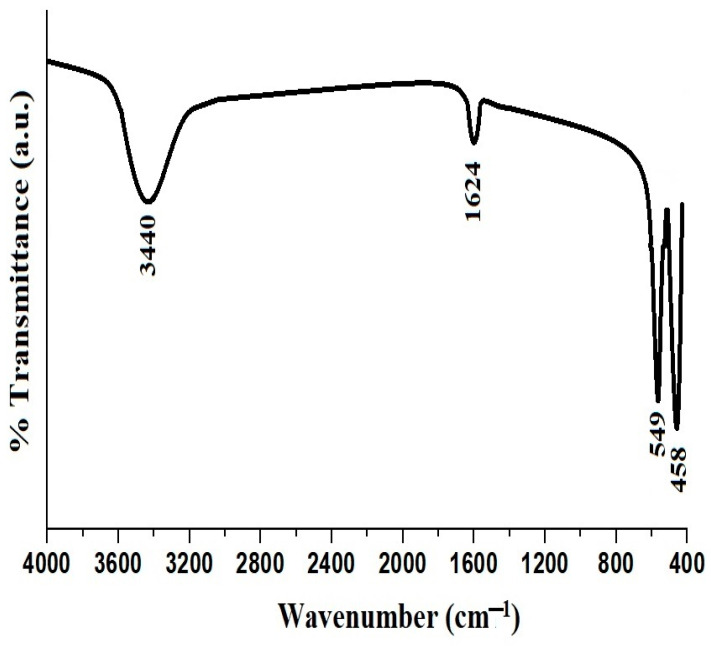
FTIR spectrum of the PF600 sample after Cd(II) adsorption.

Figure 3A represents the FTIR spectrum of the PF600 sample before adsorption, whereas Figure 9 represents the FTIR spectrum of the same sample after the adsorption of Cd(II) ions. The characteristic peaks of CuFe_2_O_4_ nanoparticles were slightly shifted, suggesting the interaction between Cd(II) ions and the surface of the nanoparticles. The absence of significant shifts in the FTIR peaks before and after adsorption suggests that the adsorption of Cd(II) ions occurs predominantly through physical interactions rather than chemical modifications of the functional groups. The interaction between the Cd(II) ions and the nanoparticles is likely due to electrostatic attractions and surface adsorption mechanisms, which do not significantly alter the vibrational frequencies of the functional groups involved.

#### 2.2.2. Influence of Contact Time

**Figure 10:**
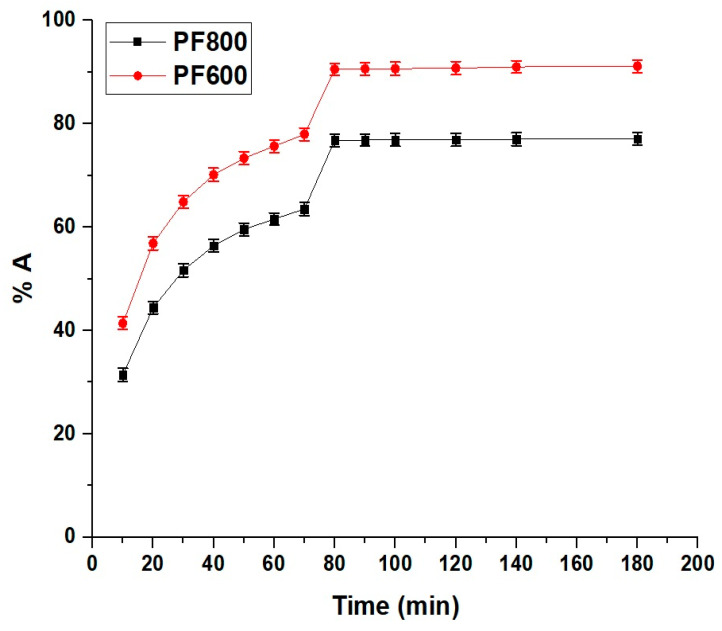
Impact of contact time on the adsorption percentage of cadmium ions by the PF800 and PF600 products.

Figure 10 clarifies how the duration of contact impacts the adsorption of cadmium ions by the PF800 and PF600 products. There is a marked increase in the percentage of Cd(II) ions adsorbed with longer contact times, which plateaus after 80 min. Hence, our findings reveal that the adsorption equilibrium is reached within 80 min, which is faster compared to other adsorbents like modified biodegradable magnetic sorbents and chitosan-alginate beads, which require longer contact times to reach equilibrium [38,39]. This rapid rise in adsorption suggests an abundance of active sites or a substantial surface area on the samples, facilitating interactions with the Cd(II) ions. Additionally, BET surface area analysis was conducted, revealing that the PF600 sample had a higher surface area of 72.27 m²/g compared to 41.16 m²/g for the PF800 sample. This substantial surface area contributes significantly to the enhanced adsorption performance of the PF600 nanoparticles. The plateau indicates that an equilibrium condition has been achieved, signifying that the adsorption sites have become fully occupied by the Cd(II) ions. Moreover, the dataset demonstrates a noticeable divergence in the adsorption percentages between the two types of nanoparticles as time progresses. Specifically, the percentages of cadmium ions adsorbed through the PF800 and PF600 products are 76.81% and 90.54%, respectively, upon reaching the 80-min mark.

##### Adsorption Kinetics

The subsequent Equations (1) and (2) are linear interpretations of the pseudo-first-order and pseudo-second-order kinetic models that have been applied to examine the Cd(II) ions’ adsorption mechanisms on the PF800 and PF600 samples [18,40,41].

(1) $$\mathrm{Pseudo}\text{-}\mathrm{first}\text{-}\mathrm{order}:\;\log\left(O_{e}-O_{t}\right)=logO_{e}-\frac{L_{1}}{2.303}t$$

(2) $$\mathrm{Pseudo}\text{-}\mathrm{second}\text{-}\mathrm{order}:\;\frac{t}{O_{t}}=\frac{1}{L_{2}O_{e}^{2}}+\frac{1}{O_{e}}t$$

where O_t_ (mg/g) represents the amount of Cd(II) ions eliminated at contact time t whereas O_e_ (mg/g) indicates the equilibrium adsorption capacity. Furthermore, the rate constants L_1_ and L_2_ signify the rate of the pseudo-first-order as well as pseudo-second-order models, with the units of 1/min and g/mg·min, respectively.

**Figure 11:**
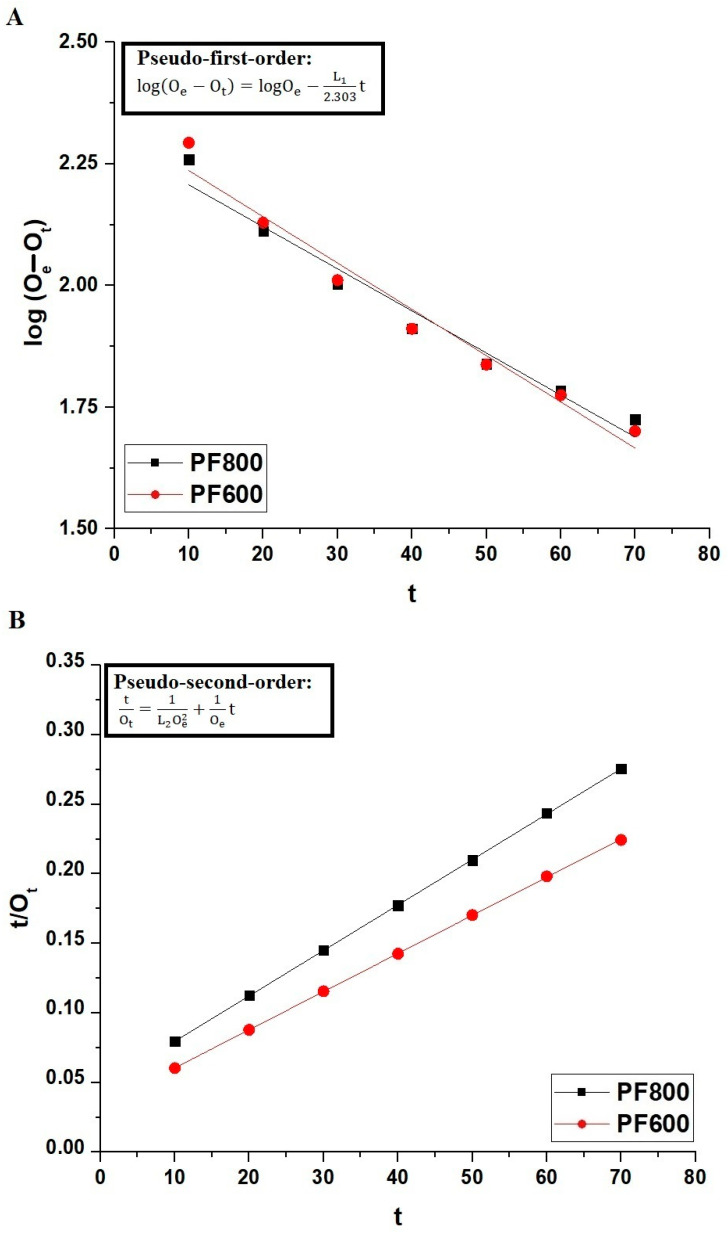
Linear kinetic modeling of Cd(II) ions adsorption onto the PF800 and PF600 products by the pseudo-first-order (**A**) as well as the pseudo-second-order (**B**) kinetic plots.

Figure 11, along with Table 1, provides a detailed examination of the kinetic behavior of cadmium ion adsorption on the PF800 and PF600 samples. The adequacy of the pseudo-first-order kinetic model is lower when compared to the pseudo-second-order kinetic model, which is reflected in the experimental data (O_Exp_) and the correlation coefficients (R^2^) indicated in Table 1. The R^2^ values for the pseudo-second-order kinetics model are closer to unity for both PF800 and PF600 samples, indicating a tighter congruence with the actual experimental data. The residual sum of square values (RSS) for the pseudo-second-order kinetics model is less than 1, indicating a tighter congruence with the actual experimental results. In addition, the calculated equilibrium capacities (O_e_) from the pseudo-second-order kinetics model align very closely with the experimental adsorption values (O_Exp_), suggesting that the adsorption process adheres more to the pattern characterized by the pseudo-second-order kinetics.

Moreover, the equations below (3) and (4) represent the nonlinear versions of both the pseudo-first-order and pseudo-second-order kinetic models that were utilized to analyze the adsorption process of Cd(II) ions on the PF800 and PF600 samples [42].

(3) $$\mathrm{Pseudo}\text{-}\mathrm{first}\text{-}\mathrm{order}:O_{t}=O_{e}\left(1-e^{-L_{1}t}\right)$$

(4) $$\mathrm{Pseudo}\text{-}\mathrm{second}\text{-}\mathrm{order}:O_{t}=\frac{O_{e}^{2}L_{2}t}{1+O_{e}L_{2}t}$$

**Figure 12:**
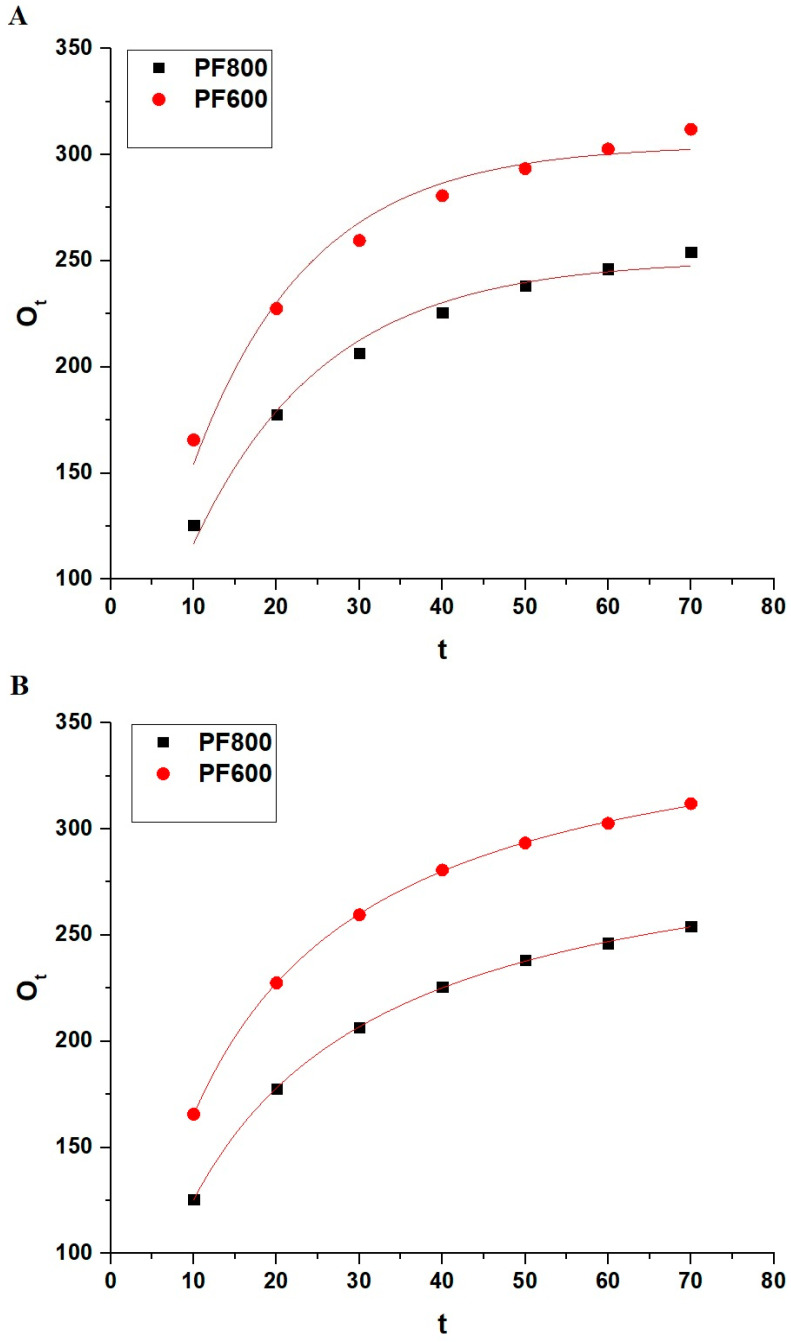
Non-linear kinetic modeling of Cd(II) ion adsorption onto the PF800 and PF600 products by the pseudo-first-order (**A**) as well as the pseudo-second-order (**B**) kinetic plots.

Figure 12 and Table 2 jointly offer an in-depth evaluation of the nonlinear models for Cd(II) adsorption on the PF800 and PF600 samples. Due to the higher R^2^ values of the non-linear pseudo-second-order model compared to the non-linear pseudo-first-order, adsorption follows the non-linear pseudo-second-order. This was confirmed by the smaller values of chi-square (χ^2^) than one in the case of the non-linear pseudo-second-order model compared to the non-linear pseudo-first-order. In addition, the calculated equilibrium capacities (O_e_) from the non-linear pseudo-second-order kinetics model align very closely with the experimental adsorption values (O_Exp_), suggesting that the adsorption process adheres more to the pattern characterized by the non-linear pseudo-second-order kinetics.

#### 2.2.3. Influence of Temperature

**Figure 13:**
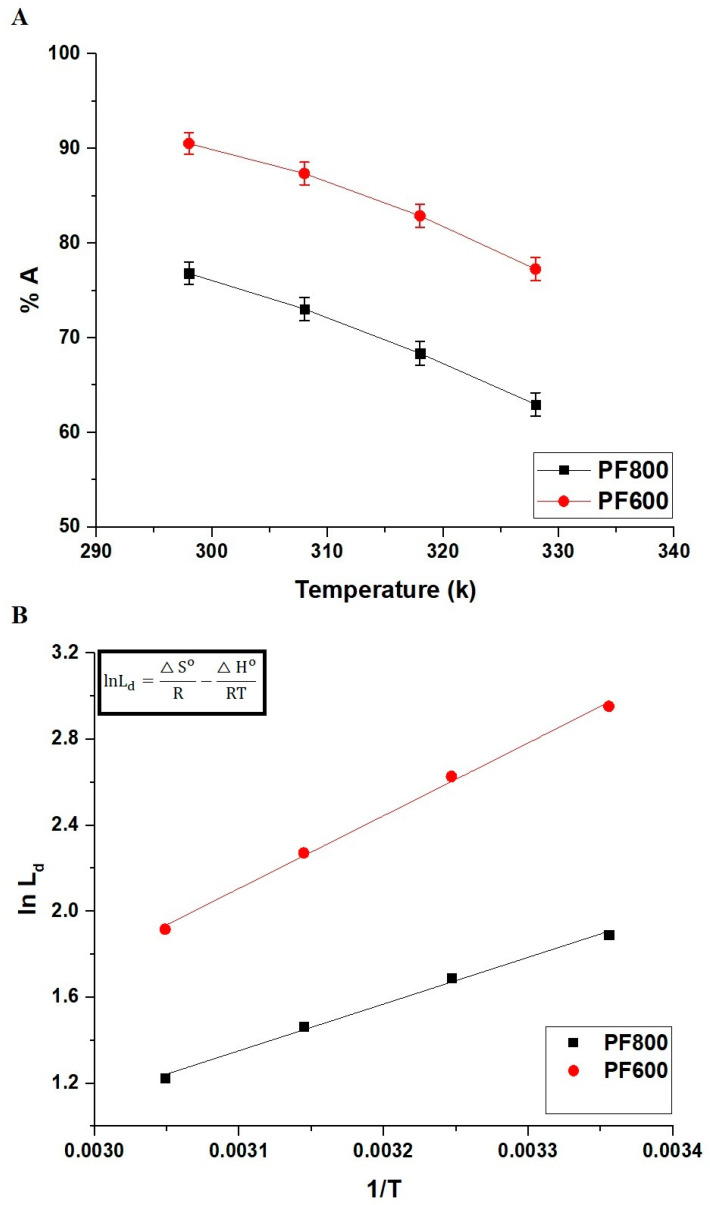
Impact of temperature on the adsorption percentage of cadmium ions by the PF800 and PF600 products (**A**). The plot of ln L_d_ versus 1/T (**B**).

Figure 13A displays how effectively the PF800 and PF600 products adsorb Cd(II) ions at various temperatures. The diminishing adsorption percentage of Cd(II) ions as the temperature increases reveals that the adsorption onto the PF800 or PF600 products is an exothermic reaction.

##### Adsorption Thermodynamics

Equations (5)–(7) were utilized to compute the Gibbs free energy (ΔG°), entropy change (ΔS°), and enthalpy change (ΔH°) for the removal of Cd(II) ions [19,40,41].

(5) $$L_{d}=\frac{O_{e}}{C_{e}}$$

(6) $$lnL_{d}=\frac{{△S}^{o}}{R}-\frac{{△H}^{o}}{RT}$$

(7) $${△G}^{o}={△H}^{o}-T{△S}^{o}$$

R is described as the gas constant (KJ/molK), T is described as the temperature (K), and L_d_ is described as the distribution coefficient (L/g).

Figure 13B and Table 3 examine the thermodynamic properties associated with the adsorption of cadmium ions by the PF800 and PF600 products. Both samples show a positive entropy change (ΔS°), suggesting increased disorder at the solution-solid interface throughout adsorption. Additionally, the negative ΔG° values at various temperatures affirm the spontaneous nature of the removal process. Moreover, the decrease in ΔH° observed in both samples supports the finding that the adsorption process is exothermic and mainly physical.

#### 2.2.4. Influence of Adsorbent Dosage

**Figure 14:**
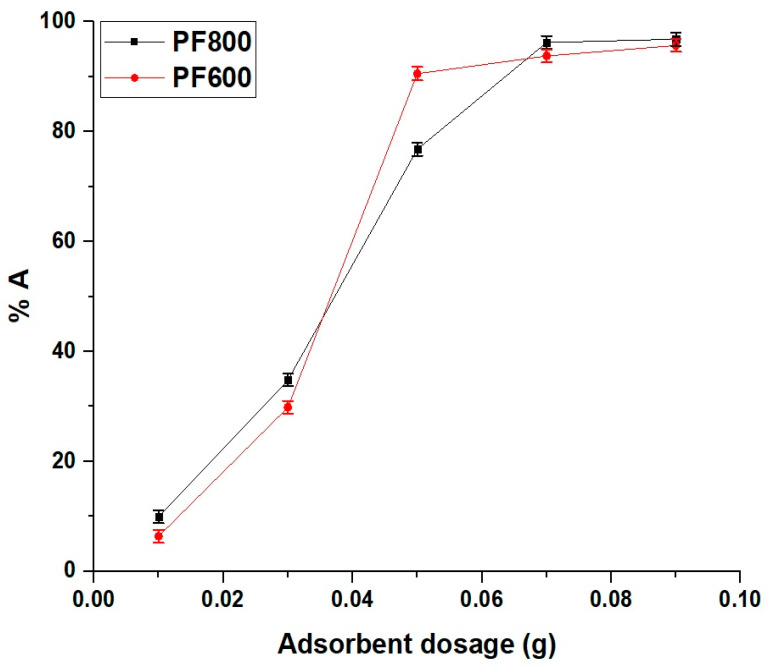
Impact of adsorbent dosage on the adsorption percentage of cadmium ions by the PF800 and PF600 products.

The results, shown in Figure 14, indicate that the adsorption efficiency of Cd(II) ions increased with the amount of adsorbent up to 0.05 g. Beyond this point, the increase in adsorption efficiency became less significant, suggesting that the remaining available adsorbent did not contribute significantly to additional adsorption. Therefore, 0.05 g was chosen as the optimal amount for further experiments to ensure a balance between efficiency and practicality.

#### 2.2.5. Influence of Concentration

**Figure 15:**
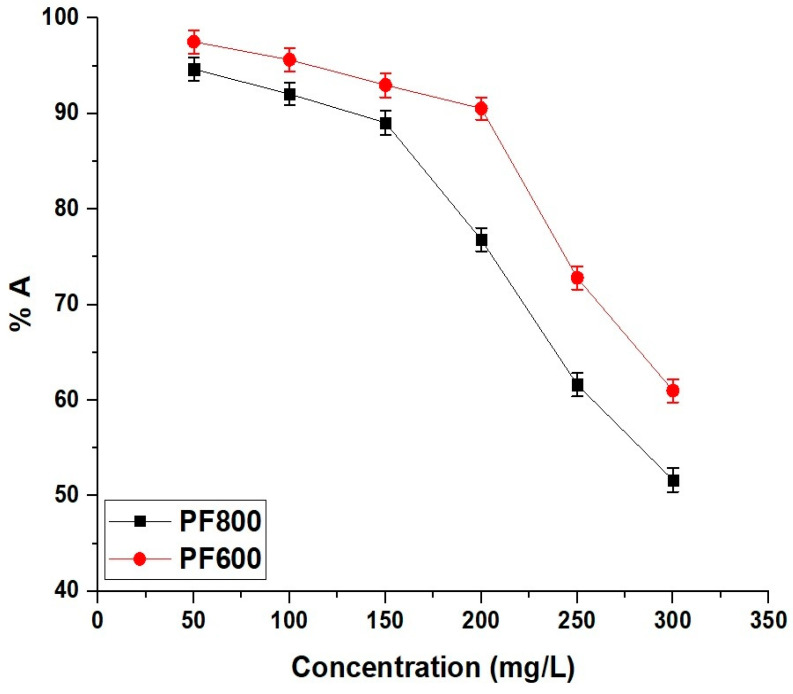
Impact of initial Cd(II) concentration on the adsorption percentage of Cd(II) ions by the PF800 and PF600 samples.

Figure 15 illustrates how the initial concentration of cadmium ions affects the adsorption efficacy of the PF800 and PF600 samples. In both samples, the adsorption percentage of cadmium ions decreases as the initial concentration increases. Also, at lower cadmium concentrations, more active sites are available relative to the number of ions, resulting in higher elimination percentages. However, as the cadmium concentration expands, these active sites become progressively occupied until they reach saturation. Once saturation is accomplished, no additional cadmium ions can be efficiently adsorbed, causing a reduction in elimination effectiveness.

##### Adsorption Isotherms

The adsorption isotherms are essential for understanding the adsorption mechanism and the interaction between the adsorbent and adsorbate. The Langmuir isotherm assumes monolayer adsorption on a homogeneous surface with a finite number of identical sites, whereas the Freundlich isotherm describes adsorption on heterogeneous surfaces with sites that have different energies of adsorption. These isotherms help to elucidate the nature of the adsorption process and the potential maximum adsorption capacity of the adsorbent. The following Equations (8) and (9) display the linear forms of the Langmuir and Freundlich equilibrium isotherms that were utilized to analyze the adsorption of Cd(II) ions onto the PF800 and PF600 samples [18,43,44].

(8) $$Freundlich\;isotherm:lnO_{e}=lnL_{3}+\frac{1}{Z}lnC_{e}$$

(9) $$Langmuir\;isotherm:\frac{C_{e}}{O_{e}}=\frac{1}{L_{4}O_{max}}+\frac{C_{e}}{O_{max}}$$

1/Z is described as the adsorption intensity. Besides, the rate constants L_4_ and L_3_ indicate the rate of the Langmuir as well as Freundlich isotherms, with the units of (L/mg) and (mg/g)(L/mg)^1/z^), respectively. Additionally, O_max_ is described as the maximum uptake capability (mg/g). Equation (10) was utilized to calculate O_max_ by employing the Freundlich equilibrium isotherm [18].

(10) $$O_{max}=L_{3}\left(C_{o}^{1/Z}\right)$$

**Figure 16:**
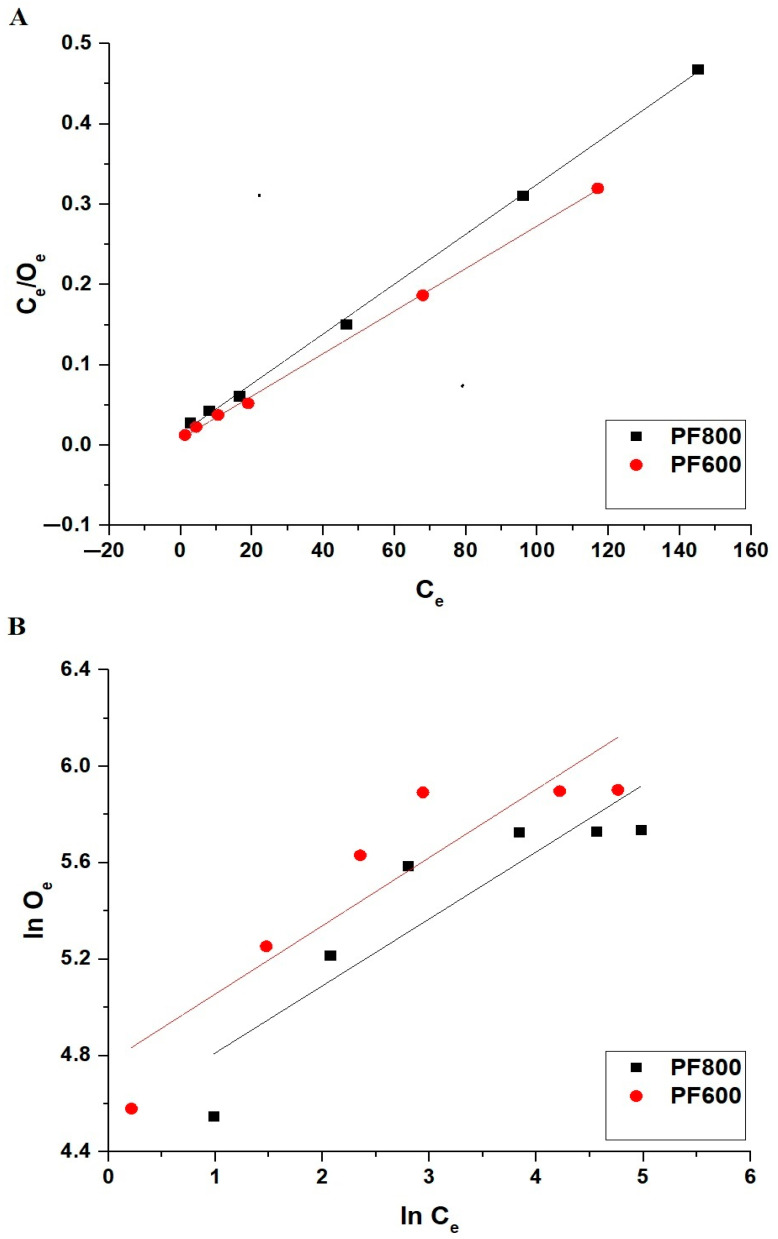
Linear equilibrium modeling of Cd(II) ion adsorption onto the PF800 and PF600 products by the Langmuir (**A**) and the Freundlich (**B**) equilibrium plots.

Figure 16 and Table 4 together provide a detailed analysis of the equilibrium conditions for the adsorption of cadmium ions onto the PF800 and PF600 products. Compared to the Langmuir plot, the Freundlich plot shows a less accurate fit, as evidenced by the correlation coefficients (R^2^) listed in Table 4. For both the PF800 and PF600 samples, the Langmuir isotherm yields R^2^ values close to 1, indicating a better match with the actual experimental results. The residual sum of square values (RSS) for the Langmuir isotherm is less than 1, indicating a tighter congruence with the actual experimental data.

Furthermore, the subsequent Equations (11) and (12) present the nonlinear representations of both the Langmuir and Freundlich equilibrium isotherms employed to examine the adsorption of Cd(II) ions onto the PF800 and PF600 products [42].

(11) $$O_{e}=\frac{O_{max}L_{4}C_{e}}{1+L_{4}C_{e}}$$

(12) $$O_{e}=L_{3}\times C_{e}^{1/Z}$$

**Figure 17:**
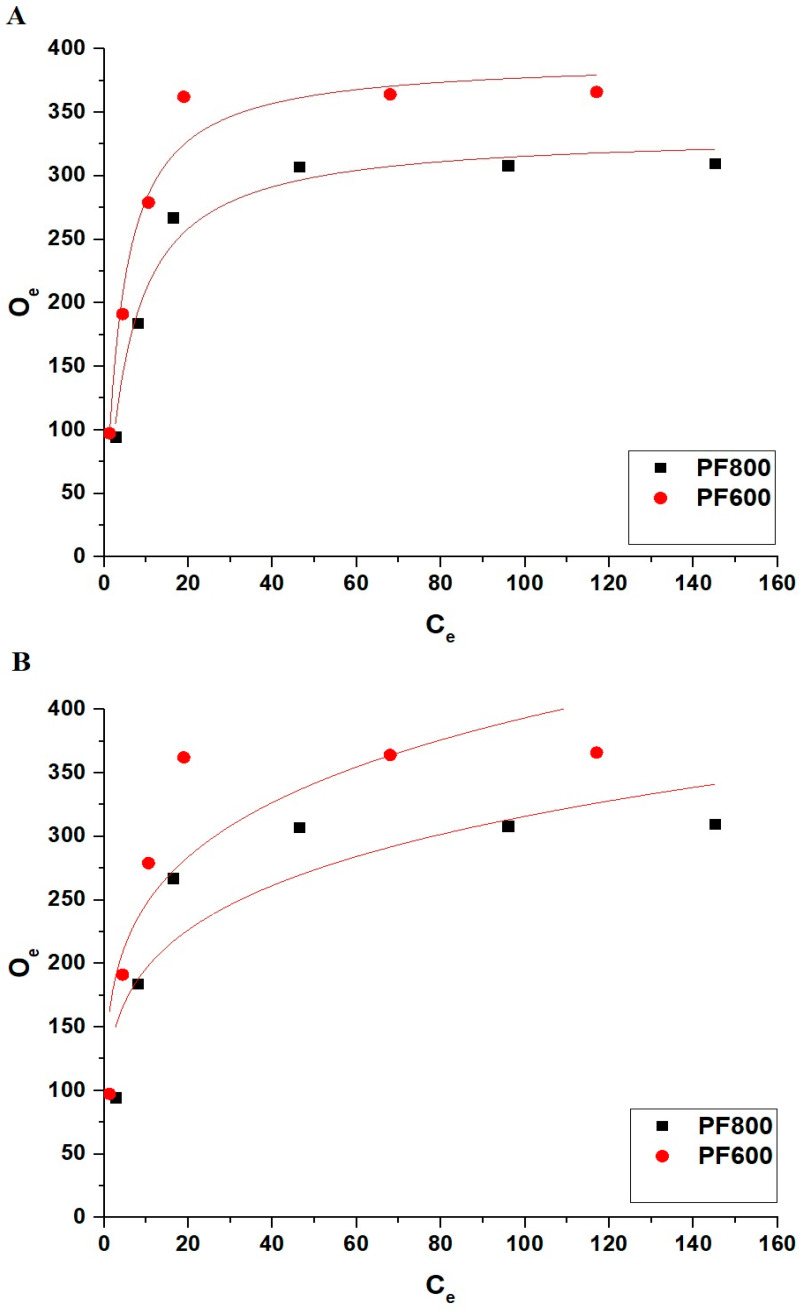
Non-linear equilibrium modeling of Cd(II) ion adsorption onto the PF800 and PF600 products by the Langmuir (**A**) as well as the Freundlich (B) equilibrium plots.

Figure 17 and Table 5 collectively offer a thorough examination of the nonlinear equilibriums concerning the adsorption of Cd(II) ions onto the PF800 and PF600 samples. Due to the low values of R^2^ and high values of χ^2^, the adsorption process does not follow non-linear models.

The data comparison of numerous adsorbents (Table 6) exhibits that the PF800 and PF600 products demonstrate superior uptake capacities of 322.58 and 377.36 mg/g, respectively, outperforming other materials [34,35,38,39,45,46]. The adsorption capacity for Cd(II) ions is higher for PF600 (377.36 mg/g) compared to PF800 (322.58 mg/g). This is attributed to the higher surface area and more active sites available in PF600 due to its smaller crystallite size and higher surface area. This comparison clearly shows that the synthesized CuFe_2_O_4_ nanoparticles exhibit significantly higher adsorption capacities compared to other adsorbents reported in the literature, such as modified biodegradable magnetic sorbent (251.88 mg/g), chitosan-alginate beads (207.00 mg/g), and others. This enhanced performance can be attributed to the unique properties of the nanoparticles synthesized via the Pechini sol-gel approach, which ensures high purity, crystallinity, and optimal particle size for maximum adsorption efficiency.

#### 2.2.6. Effect of Regeneration and Reusability

**Figure 18:**
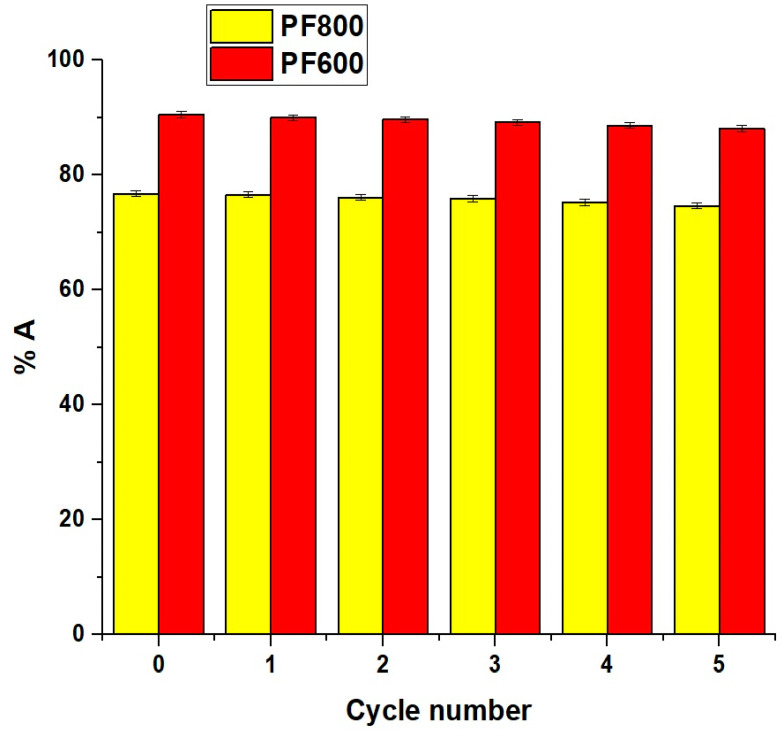
Impact of reusability of the PF800 and PF800 products for the sorption of cadmium ions.

The percentage of desorption (%D) of cadmium ions from the PF800 and PF600 adsorbents after 80 min was 99.76 and 99.86%, respectively. Times longer than 80 min does not achieve a higher desorption percentage, indicating that 80 min is sufficient for the maximum desorption percentage. EDTA (ethylenediaminetetraacetic acid) was chosen as the eluent for the desorption of Cd(II) ions due to its strong chelating properties, which allow it to form stable complexes with heavy metal ions, including Cd(II). Other eluents, such as nitric acid and hydrochloric acid, were considered, but EDTA was preferred due to its effectiveness [48]. Additionally, the regenerated PF800 and PF600 adsorbents were washed with distilled water to prepare them for further removal cycles. In addition, the influence of five sequential adsorption/desorption cycles on the adsorption of cadmium ions was investigated, with findings detailed in Figure 18. The data shows a minor reduction in the adsorption percentage of cadmium ions by the PF800 and PF600 products as the cycle count rose. Thus, it is evident that the PF800 and PF600 products display excellent reusability in capturing Cd(II) ions.

The high desorption percentages and excellent reusability of our CuFe_2_O_4_ nanoparticles over five cycles are comparable to or even better than those reported for advanced adsorbents like nano-metal oxides and MOFs (Metal-Organic Frameworks) [5,49]. The minimal reduction in adsorption efficiency over multiple cycles demonstrates the robustness and practical applicability of our synthesized nanoparticles for continuous use in water purification processes.

To confirm the stability of the adsorbents, XRD analysis was conducted on the PF600 and PF800 nanoparticles after the regeneration process (Figures omitted for brevity). The XRD patterns of the regenerated adsorbents showed no significant changes compared to the original samples, confirming that the crystal structure of the copper ferrite nanoparticles remained intact after multiple adsorption-desorption cycles.

#### 2.2.7. Selectivity of the Adsorbents

The selectivity of the adsorbents for Cd(II) ions in binary systems was assessed by comparing their adsorption capacities for Cd(II) ions in the presence of Pb(II), Zn(II), Cu(II), Ca(II), and Mg(II) ions. Batch adsorption experiments were conducted under similar conditions, and the results are summarized in Table 7. The selectivity of PF600 and PF800 adsorbents towards Cd(II) ions was not exceptionally high compared to those for other metal ions. This can be attributed to the competitive adsorption behavior of the metal ions on the surface of the adsorbents, which is influenced by factors such as ionic radius, hydration energy, and the nature of the functional groups on the adsorbent surface. The ionic radius and hydration energy of the metal ions play a significant role in their adsorption. Cd(II) ions have a relatively larger ionic radius and lower hydration energy compared to other ions like Zn(II) and Cu(II). This means that while Cd(II) ions can adsorb effectively, other ions with smaller radii and higher hydration energies can compete more effectively for the adsorption sites. The surface of the PF600 and PF800 nanoparticles contains various functional groups that interact with the metal ions. These interactions include electrostatic attractions. The affinity of these functional groups for different metal ions varies, leading to competitive adsorption.

#### 2.2.8. Stability of the PF600 and PF800 Samples under Acidic Medium

To evaluate the stability of the PF600 and PF800 adsorbents in an acidic medium, a leaching test was conducted by immersing 0.1 g of each adsorbent in 50 mL of 0.25 M HCl solution. The mixtures were stirred at room temperature, and aliquots were taken at different time intervals (0, 1, 2, 4, 8, 12, and 24 h). The concentration of iron and copper ions in the solution was determined using an atomic absorption spectrophotometer (AAS). The results showed that there were no detectable concentrations of iron and copper ions in the solutions throughout the duration of the test. This confirms that both PF600 and PF800 adsorbents are highly stable in acidic conditions, with no significant leaching of iron or copper ions even after 24 h of exposure to 0.25 M HCl.

## 3. Experimental

### 3.1. Materials

Copper(II) nitrate trihydrate (Cu(NO_3_)_2_·3H_2_O, 99.5% purity), hydrochloric acid (HCl, 37% *w*/*w*), tartaric acid (C_4_H_6_O_6_, 99% purity), iron(III) nitrate nonahydrate (Fe(NO_3_)_3_·9H_2_O, 98% purity), propylene glycol (C_3_H_8_O_2_, 99% purity), potassium chloride (KCl, 99% purity), ethylenediaminetetraacetic acid disodium salt dihydrate (C_10_H_14_N_2_Na_2_O_8_·2H_2_O, 99% purity), sodium hydroxide (NaOH, 98% purity), and cadmium(II) nitrate tetrahydrate (Cd(NO_3_)_2_·4H_2_O, 98% purity) were purchased from Sigma-Aldrich and used as received without further purification.

### 3.2. Synthesis of Copper Ferrite (CuFe_2_O_4_) Nanoparticles

**Scheme 3:**
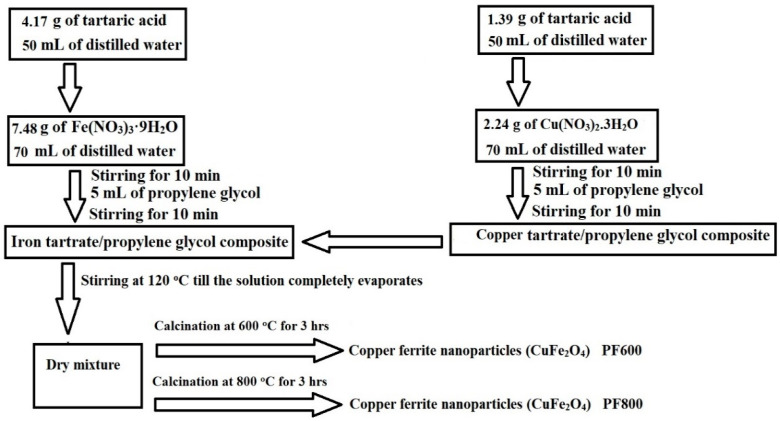
Practical steps for producing copper ferrite (CuFe_2_O_4_) nanoparticles using the Pechini sol-gel method.

Scheme 3 represents the practical steps for the production of copper ferrite (CuFe_2_O_4_) nanoparticles using the Pechini sol-gel method [27,28,29,30]. Initially, 4.17 g of tartaric acid was solubilized in 50 mL of distilled water, while separately, 7.48 g of Fe(NO_3_)_3_·9H_2_O was solubilized in 70 mL of distilled water. These solutions were combined and stirred for 10 min then 5 mL of propylene glycol was added, followed by an additional stirring period of 10 min, resulting in an iron tartarate/propylene glycol composite. Also, 1.39 g of tartaric acid was solubilized in 50 mL of distilled water, while separately, 2.24 g of Cu(NO_3_)_2_·3H_2_O was solubilized in 70 mL of distilled water. These solutions were combined and stirred for 10 min then 5 mL of propylene glycol was added, followed by an additional stirring period of 10 min, resulting in a copper tartarate/propylene glycol composite. Both metal tartarate/propylene glycol composites were then combined and stirred at 120 °C until the solution completely evaporated, leaving a dry mixture. The dry mixture was subjected to calcination in air at a heating rate of 5 °C/min with an ambient flow rate, carried out at 600 °C for 3 h to obtain PF600 and at 800 °C for 3 h to obtain PF800. After calcination, the samples were allowed to cool to room temperature naturally in the furnace.

The cooled nanoparticles were ground using agate mortar and stored in airtight containers to prevent contamination. The molar ratio of Fe(NO_3_)_3_·9H_2_O to Cu(NO_3_)_2_·3H_2_O to tartaric acid was maintained at 2:1:4 for the synthesis of copper ferrite nanoparticles.

### 3.3. Instrumentation

Infrared spectra utilizing the Fourier transform infrared (FTIR) technique for the PF600 and PF800 samples were recorded with a Thermo Fisher Nicolet IS10 spectrometer (Thermo Fisher Scientific, Waltham, MA, USA). The chemical crystalline structures of the PF600 and PF800 samples were investigated utilizing an X-ray powder diffractometer (XRD, model D8 Advanced from Bruker Co., Ltd., Karlsruhe, Germany) equipped with a Cu Kα radiation source (wavelength of 1.5406 Å) and implemented at a generator voltage and current of 40 kV and 40 mA, respectively. The surface morphology as well as the elemental constitution of the PF600 and PF800 products were derived by a JEOL JSM6360 scanning electron microscope (FE-SEM, JEOL Ltd., Tokyo, Japan)/Energy-dispersive X-ray spectrophotometer (EDX, model X-MaxN 80, Oxford Instruments, Abingdon, UK). Also, the morphology of the PF600 and PF800 products was investigated by JEM-2100Plus high-resolution transmission electron microscopy (HR-TEM, JEOL Ltd., Tokyo, Japan).

### 3.4. Removal of Cd(II) Ions from Aqueous Media

Initially, the effect of pH on the adsorption of Cd(II) ions through the PF600 and PF800 products was studied. The pH of the solution was alternated from 2.5 to 7.5 while keeping the concentration of cadmium ions at 200 mg/L, the volume of cadmium solution at 100 mL, the amount of synthesized adsorbent at 0.05 g, the contact time at 180 min, and the temperature at 298 K. This range was chosen to cover acidic to near-neutral conditions, which are commonly encountered in contaminated water sources. The effect of contact time on the adsorption of Cd(II) ions was assessed by varying the time from 10 to 180 min. During these experiments, the concentration of Cd(II) ions was maintained at 200 mg/L, the volume of cadmium solution at 100 mL, the amount of synthesized adsorbent at 0.05 g, the pH at 7.5, and the temperature at 298 K. These intervals were selected to comprehensively capture the adsorption kinetics and identify the equilibrium time. The impact of disposal temperature on the adsorption of cadmium ions was also examined by altering the temperature from 298 to 328 K. All other conditions were kept unchanged with a Cd(II) ion concentration of 200 mg/L, a volume of cadmium solution of 100 mL, an amount of synthesized adsorbent of 0.05 g, a pH of 7.5, and a contact time of 80 min. These temperatures were selected to examine the thermodynamic behavior of the adsorption process within a practical and relevant range for environmental applications. To investigate the effect of adsorbent dosage on the adsorption process of Cd(II) ions, various amounts of CuFe_2_O_4_ nanoparticles (ranging from 0.01 g to 0.09 g) were tested. The experiments were conducted under the following conditions: the concentration of Cd(II) ions was maintained at 200 mg/L, the volume of the Cd(II) solution was kept at 100 mL, the pH was adjusted to 7.5, the contact time was set to 80 min, and the temperature was maintained at 298 K. Lastly, the effect of Cd(II) concentration on adsorption was explored by varying the concentration from 50 to 300 mg/L. In these experiments, the volume of the Cd(II) solution was 100 mL, the amount of adsorbent was 0.05 g, the temperature was kept at 298 K, the pH was fixed at 7.5, and the contact time was set to 80 min. This range was chosen to understand the adsorption capacity and efficiency of the synthesized nanoparticles across various contamination levels.

**Scheme 4:**
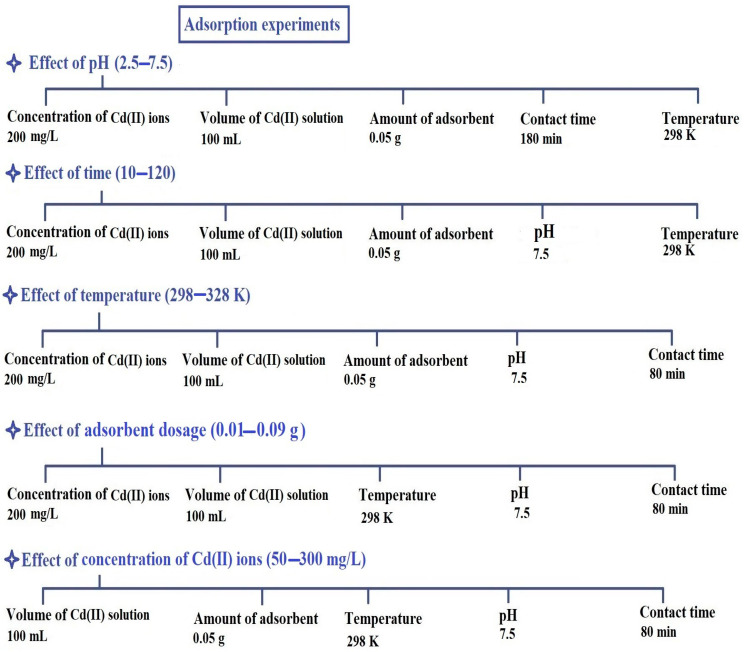
Experimental influences for studying the adsorption of Cd(II) ions by the PF600 and PF800 products.

After studying any effect, copper ferrite nanoparticles can be removed from aqueous solutions using a magnet because the synthesized CuFe_2_O_4_ nanoparticles are ferromagnetic. Scheme 4 represents the experimental influences for studying the disposal of cadmium ions by the PF600 and PF800 products.

The efficacy of the PF600 and PF800 samples to capture Cd(II) ions from the solution was assessed by determining their adsorption capacity (notated as O, in mg/g) as well as the percentage of adsorption (%A), applying Equations 13 and 14 for these respective evaluations [50,51,52,53,54,55].

(13) $$O=\left(C_{o}-C_{e}\right)\times \frac{V}{W}$$

(14) $$\%A=\frac{C_{o}-C_{e}}{C_{o}}\times 100$$

C_o_= concentration of Cd(II) ions before adsorption (mg/L) whereas C_e_= concentration of Cd(II) ions at equilibrium (mg/L). In addition, V= volume of Cd(II) solution (L) whereas W= mass of the adsorbent (g).

**Scheme 5:**
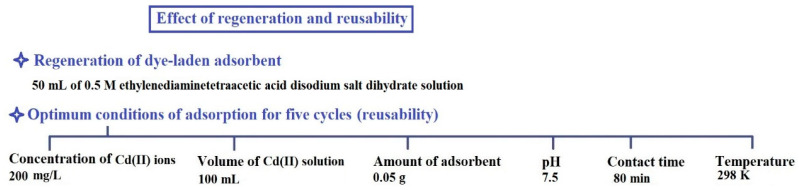
Assessing the regeneration and reusability of adsorbents in the removal of Cd(II) ions under optimal conditions.

The practical aspects of the scientific research on the regeneration and reusability of adsorbents for the removal of Cd(II) ions were meticulously investigated under optimal conditions as delineated in Scheme 5. In the process of regeneration, the dye-laden adsorbent was treated with 50 mL of a 0.5 M ethylenediaminetetraacetic acid disodium salt dihydrate solution for 80 min. This step was crucial for restoring the adsorbent’s ability to capture Cd(II) ions effectively from the solutions. The desorption percentage (%D) of Cd(II) ions was determined through Equation (15) [18,56,57].

(15) $$\%D=\frac{100C_{d}V_{d}}{(C_{o}-C_{e})V}$$

V_d_ (L) refers to the volume of the eluting agent, whereas C_d_ (mg/L) refers to the concentration of Cd(II) ions in the eluting agent.

For the assessment of reusability, the adsorbent’s performance was evaluated over five consecutive cycles. This number was selected to demonstrate the practical applicability and durability of the adsorbents over multiple uses. The conditions maintained throughout these cycles were consistent: a concentration of cadmium ions at 200 mg/L, a volume of cadmium solution at 100 mL, an amount of synthesized adsorbent at 0.05 g, a pH level at 7.5, a contact time of 80 min, and a controlled temperature of 298 K.

To evaluate the selectivity of the PF600 and PF800 copper ferrite nanoparticles for Cd(II) ions in the presence of another heavy metal ion, a series of batch adsorption experiments were conducted in binary metal systems. The metals chosen for this study were Pb(II), Zn(II), Cu(II), Ca(II), and Mg(II), which are commonly found in industrial wastewater. The initial concentrations of these metal ion solutions were set at 200 mg/L. After that, 0.05 g of the PF600 or PF800 nanoparticles were added to 100 mL of binary metal ion solutions in separate 250 mL Erlenmeyer flasks at 298 K. The pH of each solution was adjusted to 7.5. The flasks were stirred at 500 rpm for 80 min to ensure equilibrium was reached. After equilibration, the adsorbents were separated from the solutions using a magnet.

### 3.5. Point of Zero Charge (pH_PZC_) of the PF600 and PF800 Products

For each 50 mL solution, initial pH values were adjusted within the range of 2.50 to 11.50 utilizing a 0.1 M KCl solution. Adjustments to the desired pH values were made with the addition of either 0.1 M HCl or 0.1 M NaOH. Following this, 0.25 g of adsorbent was introduced to each solution, which was then subjected to 12 h of uninterrupted stirring. After the stirring process, the solutions underwent filtration to ascertain the pH of the resultant filtrate (pH_f_). The pH variation (ΔpH) was assessed by subtracting the initial pH (pH_i_) from the final pH (pH_f_). The data collected were used to construct a graph illustrating the correlation between ΔpH and the initial pH, identifying the X-axis crossing point as the adsorbent’s point of zero charge (pH_PZC_) [30,58,59,60].

## 4. Conclusions

Magnetic copper ferrite (CuFe_2_O_4_) nanoparticles were created using the Pechini sol-gel method and assessed for their ability to remove Cd(II) ions from water. The samples, which were synthesized at 600 °C and 800 °C, were abbreviated as PF600 and PF800, respectively. The synthesized samples were characterized using FTIR, XRD, FE-SEM, HR-TEM, and EDX, which confirmed the successful formation of CuFe_2_O_4_ spinel structures with crystallite sizes of 22.64 nm for PF600 and 30.13 nm for PF800. FE-SEM analysis revealed particle diameters of 154.98 nm for PF600 and 230.05 nm for PF800, showing spherical and irregular shapes, while HR-TEM analysis confirmed the presence of aggregated nanoparticles with average diameters of 52.26 nm for PF600 and 98.32 nm for PF800. The PF600 and PF800 nanoparticles demonstrated outstanding adsorption capacities of 377.36 mg/g and 322.58 mg/g, respectively, far surpassing many materials documented in the literature. The adsorption process adhered to the Langmuir isotherm model and pseudo-second-order kinetics, indicating monolayer adsorption and strong physical interactions. This process was spontaneous and exothermic. Optimal adsorption conditions were determined to be at a pH of 7.5, with an adsorbent dosage of 0.05 g and a contact time of 80 min at room temperature. Reusability tests indicated high adsorption efficiency across multiple cycles when desorbed with a 0.5 M ethylenediaminetetraacetic acid (EDTA) solution, underscoring the practical potential of these nanoparticles. The intrinsic magnetic properties of CuFe_2_O_4_ allowed for easy separation from the aqueous medium using a magnet, facilitating efficient and cost-effective recovery of the adsorbent.

## Figures and Tables

**Table 1:** Linear kinetic parameters for cadmium ion removal by the PF800 and PF600 products.

Samples	O_Exp_ (mg/g)	Pseudo-First-Order				Pseudo-Second-Order			
		L_1_ (1/min)	O_e_ (mg/g)	R^2^	RSS	L_2_ (g/mg.min)	O_e_ (mg/g)	R^2^	RSS
PF800	307.24	0.0199	196.53	0.9609	0.00703	0.000228	305.81	0.9999	0.00327
PF600	362.16	0.0219	214.43	0.9635	0.00794	0.000230	364.96	0.9999	0.00274

**Table 2:** Non-linear kinetic parameters for cadmium ion removal by the PF800 and PF600 products.

Samples	O_Exp_ (mg/g)	Pseudo-First-Order				Pseudo-Second-Order			
		L_1_ (1/min)	O_e_ (mg/g)	R^2^	χ^2^	L_2_ (g/mg.min)	O_e_ (mg/g)	R^2^	χ^2^
PF800	307.24	0.0628	250.64	0.9823	36.87	2.27 × 10^−4^	306.21	0.9999	0.22563
PF600	362.16	0.0707	304.59	0.9739	69.22	2.28 × 10^−4^	364.39	0.9998	0.4998

**Table 3:** Thermodynamic constants for Cd(II) ion elimination by the PF800 and PF600 products.

Samples	ΔS° (KJ/molK)	ΔH° (KJ/mol)	ΔG° (KJ/mol)			
			298	308	318	328
PF800	0.0449	−18.16	−31.56	−32.01	−32.46	−32.91
PF600	0.0699	−28.26	−49.12	−49.82	−50.52	−51.22

**Table 4:** Linear equilibrium constants for cadmium ion removal by the PF800 and PF600 products.

Samples	Langmuir				Freundlich				
	O_max_ (mg/g)	L_4_ (L/mg)	R^2^	RSS	O_max_ (mg/g)	L_3_ (mg/g)(L/mg)^1/n^	1/Z	R^2^	RSS
PF800	322.58	0.2135	0.9989	1.29 × 10^−4^	406.11	93.11	0.2779	0.7680	0.2074
PF600	377.36	0.3239	0.9991	5.56 × 10^−5^	529.85	118.28	0.2830	0.7848	0.2400

**Table 5:** Non-linear equilibrium constants for cadmium ion removal by the PF800 and PF600 products.

Samples	Langmuir				Freundlich				
	O_max_ (mg/g)	L_4_ (L/mg)	R^2^	χ^2^	O_max_ (mg/g)	L_3_ (mg/g)(L/mg)^1/n^	1/Z	R^2^	χ^2^
PF800	333.72	0.1719	0.9715	221.36	364.39	122.08	0.2064	0.7460	1974.51
PF600	391.84	0.2555	0.9616	478.57	452.48	154.96	0.2023	0.7286	3382.44

**Table 6:** Comparison of maximum adsorption capacities for various adsorbents towards Cd(II) ions including the new PF800 and PF600 products.

Adsorbent	O_max_ (mg/g)	Ref
Cerium-doped activated carbon	5.88	[46]
Polyaniline grafted chitosan	14.33	[35]
Copper ferrite/sepiolite/graphene oxide composite	19.01	[47]
Nickel ferrite	21.11	[22]
Polyethyleneimine	22.73	[45]
Nickel ferrite/graphene oxide composite	74.62	[23]
Cobalt ferrite/graphene oxide composite	105.26	[23]
Chitosan-Coated perlite beads	178.6	[34]
Chitosan-alginate beads	207.00	[39]
Modified biodegradable magnetic sorbent	251.88	[38]
PF800	322.58	This study
PF600	377.36	This study

**Table 7:** Adsorption capacities (mg/g) of PF600 and PF800 for Cd(II) ions in binary systems with other metal ions.

Binary System	Adsorbent	Cd(II)	Pb(II)	Zn(II)	Cu(II)	Ca(II)	Mg(II)
Cd(II)-Pb(II)	PF600	275.02	102.34	----	----	----	----
	PF800	229.02	93.56	----	----	----	----
Cd(II)-Zn(II)	PF600	287.24	----	90.12	----	----	----
	PF800	242.04	----	80.54	----	----	----
Cd(II)-Cu(II)	PF600	266.82	----	----	110.54	----	----
	PF800	216.35	----	----	106.23	----	----
Cd(II)-Ca(II)	PF600	207.13	----	----	----	170.23	----
	PF800	178.91	----	----	----	143.67	----
Cd(II)-Mg(II)	PF600	214.13	----	----	----	----	163.23
	PF800	184.35	----	----	----	----	138.23

## Data Availability

Data are contained within the article.
